# Hyperosmotic Stress Induces the Expression of Organic Osmolyte Transporters in Porcine Intestinal Cells and Betaine Exerts a Protective Effect on the Barrier Function

**DOI:** 10.3390/biomedicines12102391

**Published:** 2024-10-18

**Authors:** Elena De Angelis, Paolo Borghetti, Benedetta Passeri, Valeria Cavalli, Luca Ferrari, Melania Andrani, Paolo Martelli, Roberta Saleri

**Affiliations:** Department of Veterinary Science, University of Parma, Strada del Taglio 10, 43126 Parma, Italy; elena.deangelis@unipr.it (E.D.A.); paolo.borghetti@unipr.it (P.B.); benedetta.passeri@unipr.it (B.P.); valeria.cavalli@unipr.it (V.C.); luca.ferrari@unipr.it (L.F.); paolo.martelli@unipr.it (P.M.); roberta.saleri@unipr.it (R.S.)

**Keywords:** porcine intestinal cells, hyperosmotic stress, osmolyte transporters, betaine, tight junctions, cytokines

## Abstract

**Background/objectives:** The porcine intestinal epithelium plays a fundamental role as a defence interface against pathogens. Its alteration can cause severe inflammatory conditions and diseases. Hyperosmotic stress under physiological conditions and upon pathogen challenge can cause malabsorption. Different cell types counteract the osmolarity increase by accumulating organic osmolytes such as betaine, taurine, and myo-inositol through specific transporters. Betaine is known for protecting cells from hyperosmotic stress and has positive effects when fed to pigs. The aim of this study is to demonstrate the modulation of osmolyte transporters gene expression in IPEC-J2 during osmolarity changes and assess the effects of betaine. **Methods:** IPEC-J2 were seeded in transwells, where differentiate as a polarized monolayer. Epithelial cell integrity (TEER), oxidative stress (NO) and gene expression of osmolyte transporters, tight junction proteins (TJp) and pro-inflammatory cytokines were evaluated. **Results:** Cells treated with NaCl hyperosmolar medium (500 mOsm/L) showed a TEER decrease at 3 h and detachment within 24 h, associated with an osmolyte transporters reduction. IPEC-J2 treated with mannitol hyperosmolar medium (500 mOsm/L) upregulated taurine (*TauT*), myo-inositol (*SMIT*) and betaine (*BGT1*) transporters expression. A decrease in TJp expression was associated with a TEER decrease and an increase in *TNFα*, *IL6*, and *IL8*. Betaine could attenuate the hyperosmolarity-induced reduction in TEER and TJp expression, the NO increase and cytokines upregulation. **Conclusions:** This study demonstrates the expression of osmolyte transporters in IPEC-J2, which was upregulated upon hyperosmotic treatment. Betaine counteracts changes in intracellular osmolarity by contributing to maintaining the epithelial barrier function and reducing the inflammatory condition. Compatible osmolytes may provide beneficial effects in therapies for diseases characterized by inflammation and TJp-related dysfunctions.

## 1. Introduction

The gastrointestinal tract not only provides nutrient absorption but also represents the largest interface between the host and the environment and the first line of defence against harmful components and pathogens as a first site of communication with the immune system [1,2], thus playing a critical role in the growth and health of animals [3]. Firstly, the layer of intestinal epithelial cells acts as a mechanical barrier. Depending on the lumen content and the different intestine segment, intestinal epithelial cells are physiologically exposed to a change in the osmolarity of the lumen content; indeed, the osmolarity of chyme ranges from 545 to 585 mOsm/L [4]. Consequently, epithelial cells can adapt to a hyperosmotic environment compared to plasma osmolarity. Moreover, it is worth noting that not only bacteria/bacterial products but also stressful factors such as hypoxia, acidosis, and hyperosmolarity can induce intestinal cells to produce inflammatory cytokines. It was demonstrated that the hyperosmotic exposure of intestinal cells activates inflammatory cytokines such as *IL8* [5,6]. All these factors may contribute to triggering and sustaining the development and worsening of intestinal diseases [7,8].

Furthermore, when intestinal epithelial cells are infected by a virus, altered cellular metabolism and functions are thought to be responsible for abnormal absorption and secretion. The impaired digestion and absorption lead to the accumulation of lactose and carbohydrates that undergo fermentation by bacteria, thus exacerbating the diarrhea because of increased osmotic pressure [9]. Since the intestines of young animals are not fully developed, intestinal infections cause lesions by increasing intestinal permeability and the inflammation of the gut; in these conditions, intestinal cells suffer from hyperosmotic stress, which can lead to malabsorption and reduction in growth performances [10,11]. Betaine (N-trimethylglycine) is a major biocompatible osmolyte involved in cellular volume homeostasis as well as in cellular protection against oxidative stress [12] and different injuries, due to its ability to stabilize native protein structures [13,14].

Since the osmolyte uptake system of intestinal epithelial cells has not been thoroughly investigated, the aim of this study is to demonstrate the modulation of the gene expression of osmolyte (betaine, taurine, and myo-inositol) transporters in porcine intestinal epithelial cells (IPEC-J2) during osmolarity changes (500 mOsm/L) obtained using sodium chloride (NaCl) and mannitol (i.e., an osmoactive sugar poorly absorbed by intestinal cells [15]). In addition, the effect of betaine on cytokine and TJp gene expression was assessed, aiming to improve the knowledge regarding the mechanisms of osmoprotection in intestinal epithelial cells.

This could be important in enhancing the knowledge regarding the mechanisms of action of osmolytes, to use in therapies for intestinal diseases characterized by inflammation, diarrhea, and diseases inducing barrier dysfunctions through the modification of TJ proteins.

## 2. Materials and Methods

### 2.1. Media Formulation

The standard medium for IPEC-J2 cells was composed of Dulbecco’s Modified Eagle Medium/Ham’s F-12 (1:1 DMEM/Ham’s F-12) (Merck, Darmstadt, Germany) with 5% fetal bovine serum (FBS) (ThermoFisher, Carlsbad, CA, USA), supplemented with 1% penicillin/streptomycin/amphotericin B and 2 mM glutamine (Merck, Darmstadt, Germany). The osmolarity of the culture medium was adjusted from an iso-osmotic condition (286 ± 1.0 mOsm/L) to hyperosmotic conditions with the addition of NaCl (490 ± 3.2 mOsm/L) or mannitol (482 ± 2.9 mOsm/L). Medium osmolarity was measured with a vapour pressure osmometer (Wescor 5500, Logan, UT, USA).

### 2.2. IPEC-J2 Culture Conditions

Intestinal porcine epithelial cells J2 (IPEC-J2), a non-transformed cell line derived from the jejunum of a newborn piglet (kindly provided by Dr. Baldi A. of the Department of Veterinary Science for Health, Animal Production and Food Safety, University of Milan, Lodi, Italy), were cultured in flasks in the standard medium at 37 °C, 5% CO_2_. The number of IPEC-J2 was quantified by a haemocytometer, and viability was evaluated by Trypan blue exclusion (Merck, Darmstadt, Germany). The cells between passages 28 and 30 were used.

### 2.3. Epithelial Cell Integrity Measurement

IPEC-J2 were trypsinized and seeded in transwell cell culture inserts (0.3 cm^2^ polyethylene terephthalate membrane with 0.4 μm pore size) (Costar, Corning Inc., Corning, NY, USA) at a density of 7.5 × 10^4^ cells/insert. In these conditions, IPEC-J2 undergo spontaneous differentiation, forming a polarized monolayer and exerting a barrier function through TJ formation [16].

The culture medium was changed every two days until cell confluency, and changed every day when cells reached 100% confluency/differentiation in order to ensure a stabilized culture (transepithelial electrical resistance [TEER] > 1000 Ω/cm^2^). The TEER value was determined using an EVOM2 Voltohmmeter (World Precision Instruments, Sarasota, FL, USA) with correction for basal TEER from the transwell membrane without cells. Subsequently, the medium was changed, and the experimental groups were defined as follows:

1. IPEC-J2 in DMEM/Ham’s F-12 medium (group: C)
2. IPEC-J2 in DMEM/Ham’s F-12 with betaine (5 mM) (group: C + BET)
3. IPEC-J2 in DMEM/Ham’s F-12 with NaCl (group: NaCl)
4. IPEC-J2 in DMEM/Ham’s F-12 with mannitol (group: Mann)
5. IPEC-J2 in DMEM/Ham’s F-12 with NaCl and betaine (5 mM) (group: NaCl + BET)
6. IPEC-J2 in DMEM/Ham’s F-12 with mannitol and betaine (5 mM) (group: Mann + BET)

TEER of each monolayer was measured at 10 min, 30 min, 3 h, 6 h and 24 h. All cultures were incubated at 37 °C, 5% CO_2_.

### 2.4. Griess Assay for Nitric Oxide Production

Nitrite (NO_2_^−^) in the cell culture supernatant, which is a stable indicator of nitric oxide (NO) production, was quantified by a Griess assay. All media were transferred into 96-well plates, and absorbance was read at 540 nm by using a Victor^®^ Nivo™ Multimode Microplate Reader (PerkinElmer, Waltham, MA, USA), as previously reported [17]. The concentration of NO_2_^−^ was quantified based on a nitrite (NaNO_2_) standard curve.

### 2.5. RNA Extraction and Reverse Transcription

Total RNA was extracted by using a EuroGold Trifast™ kit (Euro-clone, Milan, Italy) according to the manufacturer’s instructions and reverse-transcribed to cDNA by using oligo-dT primers (Bioneer, Daejeon, Republic of Korea) as previously reported [17]. Purity and concentration were quantified by using a BioSpectrometer^®^ (Eppendorf AG, Hamburg, Germany). RNA samples were treated with DNase (Merck; Darmstadt, Germany) and 1 µg RNA/20 µL diethylpyrocarbonate (DEPC) water was reverse-transcribed using HiScript^®^ III RT SuperMix (Vazyme Biotech Co., Nanjing, China). RT was carried out using a StepOne™ thermocycler (Applied Biosystems, Foster City, CA, USA, StepOne™ software v.2.3) according to the HiScript^®^ III RT SuperMix manufacturer’s thermal conditions, which are as follows: 2 min at 45 °C, 15 min at 37 °C, 5 s at 85 °C. cDNA samples were stored at −20 °C before being used for quantitative PCR.

### 2.6. Quantitative PCR

cDNA was used for real-time quantitative PCR (qPCR) carried out by using a StepOne™ thermocycler (Applied Biosystems, StepOne™ software v.2.3) as reported in a previous study [17]. cDNA (20 ng) was amplified by using a Fast PowerUp™ SYBR™ Green Master Mix (Applied Biosystems) and the primers (Eurofins Genomics, Ebersberg, Germany) reported in Table 1 at 300 nM for all genes except for *ZO-1* (400 nM). Reaction efficiencies ranged between 98.5% and 103.8%, slope values ranged between −3.36 and −3.03, and r^2^ values ranged between 0.98 and 0.99. Samples were maintained for 20 s at 95 °C and subjected to 40 cycles of denaturation for 3 s at 95 °C, followed by fast annealing/extension for 30 s at 60 °C. Data were evaluated using the 2^−ΔΔCt^ method [18], in which gene expression levels were normalized to the endogenous reference gene *18S rRNA* [19]. Due to the lowest variation, *18S rRNA* gene was chosen among other tested genes (i.e., *GAPDH*, *β-2MG*, and *HPRT*). The cDNA quantity was expressed as relative quantity (RQ) and calculated in relation to the expression level in IPEC-J2 incubated in control medium (C) for 3 h. A melting curve analysis (from 60 °C to 95 °C) was performed to check the specific amplification. No-RT and no-template controls were included in each run.

### 2.7. Statistical Analysis

Each experiment was repeated three times, and six replicates were used for each culture condition. ANOVA (IBM^®^ SPSS^®^ Statistics v.28, New York, NY, USA) was employed using a model with “groups” and “interaction between times” as fixed factors. When significant differences (*p* < 0.05) were found, the LSD post hoc test was used to compare means. The results are shown as means ± standard deviations. Significant differences between groups were considered for *p* < 0.05.

## 3. Results

### 3.1. Effect of NaCl Hyperosmolar Medium on TEER and Osmolyte Transporters Gene Expression

The IPEC-J2 cells seeded in transwells reached stability at 9 days, with values of TEER around 1000 ± 21 Ω/cm^2^. The treatment of stabilized cells with the hyperosmolar medium at 500 mOsm/L with NaCl caused a rapid decrease in TEER values at 3 h of treatment to 250 Ω/cm^2^ (75% reduction) and after 6 h of treatment to 125 Ω/cm^2^ (87.5% reduction) (*p* < 0.05); at 24 h, an extensive impairment of the cells with subsequent cell death and detachment was observed. The addition of the compatible osmolyte betaine did not support cell adaptation to the hyperosmotic stress.

**Figure 1:**
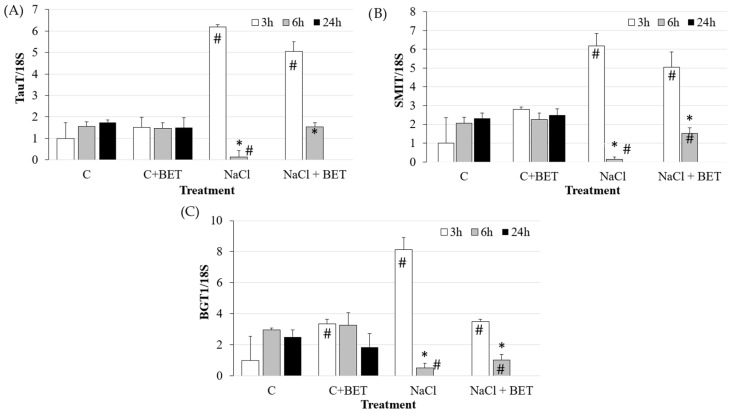
Gene expression of (**A**) *TauT*, (**B**) *SMIT* and (**C**) *BGT1* in IPEC-J2 cells cultured for the indicated time points in the control medium (C) and in the hyperosmolar medium (500 mOsm/L) obtained with NaCl without/with betaine (BET). Gene expression was measured by using RT-qPCR and normalized to that of the reference gene *18S rRNA*. Data are presented as means  ±  SD of three independent experiments, each performed in duplicate. # hashtags indicate a statistical difference between each treatment and control (C) at the same time point (*p* < 0.05); * asterisks indicate a statistical difference between each time point and 3 h upon the same treatment (*p* < 0.05).

The gene expression analysis of osmolyte transporters (Figure 1) showed that hyperosmotic stress induced a significant increase in the expression of *TauT*, *SMIT* and *BGT1* transporters at 3 h, which drastically decreased at 6 h (*p* < 0.05). At 24 h, it was not possible to carry out any analysis because of the aforementioned detrimental effect of the hyperosmotic medium. Betaine supplementation slightly attenuated the expression reduction at 6 h (*p* < 0.05).

### 3.2. Effect of Mannitol Hyperosmolar Medium on TEER and Osmolyte Transporters Gene Expression

The cell treatment with the hyperosmolar medium at 500 mOsm/L with mannitol significantly reduced TEER both at 6 h and 24 h (Table 2) (*p* < 0.05); the addition of betaine prevented the decrease observed at 6 h but not at 24 h (*p* < 0.05).

Hyperosmolar treatment with mannitol (Mann) induced a significant increase in NO release at 10 min and 30 min compared to the control medium (C) (*p* < 0.05). Betaine supplementation reduced NO release both in the control condition (C + BET) and in the hyperosmolar medium (Mann + BET) (*p* < 0.05). Also, the increase in NO levels observed upon hyperosmolar treatment was reduced as early as 3 h after betaine supplementation (*p* < 0.05) (Table 3).

**Figure 2:**
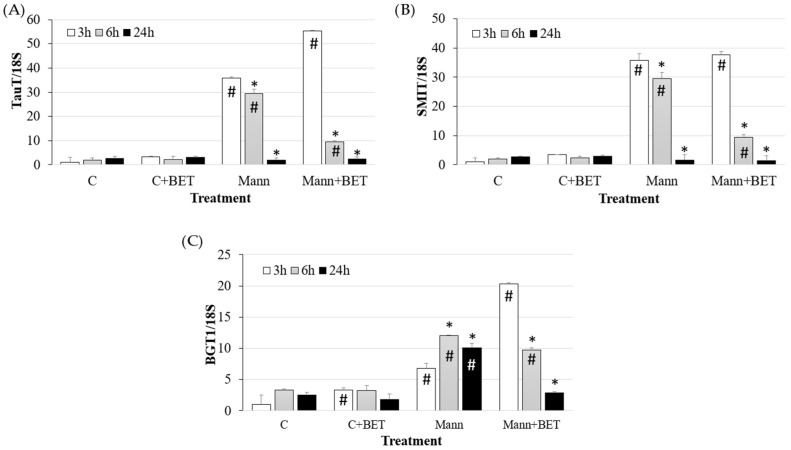
Gene expression of (**A**) *TauT*, (**B**) *SMIT* and (**C**) *BGT1* in IPEC-J2 cells cultured for the indicated time points in the control medium (C) and in the hyperosmolar medium (500 mOsm/L) obtained with mannitol (Mann) without/with betaine (BET). Gene expression was measured by using RT-qPCR and normalized to that of the reference gene *18S rRNA*. Data are presented as means  ±  SD of three independent experiments, each performed in duplicate. # hashtags indicate a statistical difference between each treatment and control (C) at the same time point (*p* < 0.05); * asterisks indicate a statistical difference between each time point and 3 h upon the same treatment (*p* < 0.05).

Under the 500 mOsm/L hyperosmolar condition, all three osmolyte transporters were induced at 3 h (*p* < 0.05) (Figure 2).

The gene expression of *TauT* and *SMIT* transporters progressively decreased and reached control values at 24 h (*p* < 0.05). The presence of betaine induced a quicker decrease in the two osmolyte transporters within 6 h (*p* < 0.05).

*BGT1* transporter gene expression remained higher than the control at 24 h (*p* < 0.05). The addition of betaine to the hyperosmolar medium induced a faster (3 h) and higher increase in *BGT1* transporter compared to the medium without betaine (*p* < 0.05) (Figure 2).

### 3.3. Effect of Mannitol Hyperosmolar Medium on Cytokine and TJp Gene Expression

**Figure 3:**
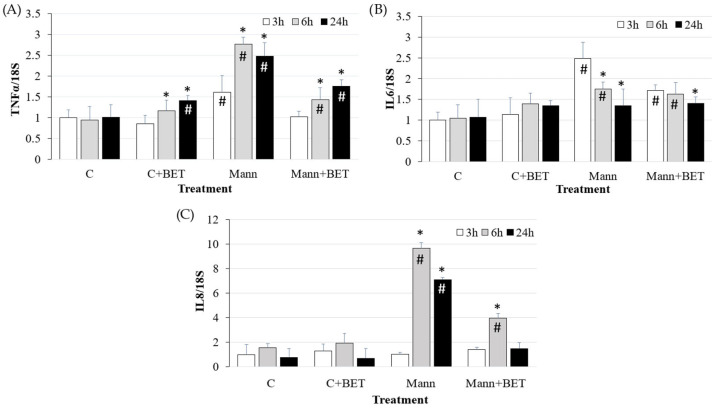
The gene expression of (**A**) *TNFα*, (**B**) *IL6* and (**C**) *IL8* in IPEC-J2 cells cultured for the indicated time points in the control medium (C) and in the hyperosmolar medium (500 mOsm/L) obtained with mannitol (Mann) without/with betaine (BET). Gene expression was measured by using RT-qPCR and normalized to that of the reference gene *18S rRNA*. Data are presented as means  ±  SD of three independent experiments, each performed in duplicate. # hashtags indicate a statistical difference between each treatment and control (C) at the same time point (*p* < 0.05); * asterisks indicate a statistical difference between each time point and 3 h upon the same treatment (*p* < 0.05).

Cells cultured in the hyperosmolar medium (500 mOsm/L mannitol) showed an increase in gene expression of the pro-inflammatory cytokines *TNFα*, *IL6*, and *IL8* compared to cells in the control medium with different kinetics (*p* < 0.05) (Figure 3).

Both *TNFα* and *IL6* expression increased at 3 h (*p* < 0.05); *TNFα* further increased at 6 h and 24 h, whereas *IL6* decreased at the same time points (*p* < 0.05).

*IL8* expression showed a 10-fold increase at 6 h and remained at high levels, including at 24 h (*p* < 0.05). The treatment with the compatible osmolyte betaine reduced *TNFα* and *IL8* expression at 6 h and 24 h, and *IL6* at 3 h (*p* < 0.05).

**Figure 4:**
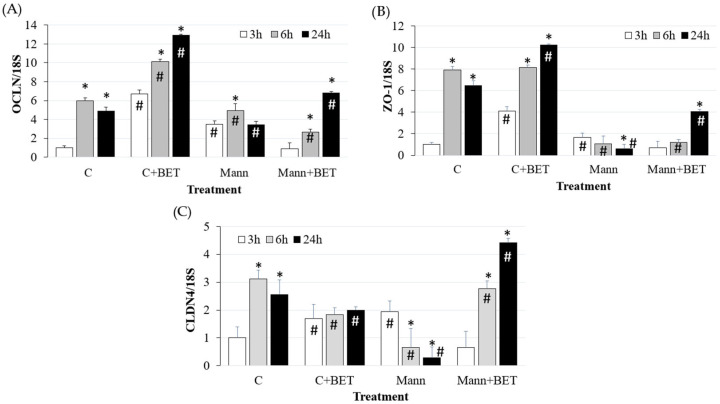
The gene expression of (**A**) *OCLN*, (**B**) *ZO-1* and (**C**) *CLDN4* in IPEC-J2 cells cultured for the indicated time points in the control medium (C) and in the hyperosmolar medium (500 mOsm/L) obtained with mannitol (Mann) without/with betaine (BET). Gene expression was measured by using RT-qPCR and normalized to that of the reference gene *18S rRNA*. Data are presented as means  ±  SD of three independent experiments, each performed in duplicate. # hashtags indicate a statistical difference between each treatment and control (C) at the same time point (*p* < 0.05); * asterisks indicate a statistical difference between each time point and 3 h upon the same treatment (*p* < 0.05).

The treatment with betaine in control cells induced a significant increase in *OCLN* and *ZO-1* expression during the 24 h period, while *CLDN4* expression increased at 3 h only (*p* < 0.05) and remained stable over time compared to the control condition (i.e., without any supplement) (Figure 4).

The hyperosmotic condition with 500 mOsm/L mannitol had a strong modulatory effect on *ZO-1* and *CLDN4* by reducing their levels at 6 h and 24 h (*p* < 0.05). The addition of betaine increased the expression levels of *OCLN* and *ZO-1* at 24 h, while *CLDN4* expression levels were upregulated at 6 h and 24 h (*p* < 0.05).

## 4. Discussion

IPEC-J2 cells are an ideal tool for studying intestinal trans-epithelial transport, interactions with enteric pathogens and the effect of nutrients on a variety of widely used parameters (e.g., transepithelial electrical resistance, permeability, metabolic activity) to evaluate the functions and effectiveness of the epithelial barrier [16].

In this study, we evaluated the effects of a hyperosmolar treatment on in vitro stabilized IPEC-J2 porcine intestinal cells. For testing whether these cells respond to a hyperosmolarity through the induction of osmolyte transporters, IPEC-J2 were seeded in transwells, where they spontaneously differentiated by forming a polarized monolayer and exerting a barrier function through TJ formation, which better mimics in vivo conditions [16].

The data showed that the use of NaCl to induce hyperosmolarity caused a decrease in TEER values at 3 h of treatment and the detachment of cells from transwells within 24 h.

When hyperosmolar stress is obtained by adding NaCl, intracellular ionic strength increases. However, in addition to augmented tonicity, high Na^+^ and Cl^−^ ion concentrations may have specific effects on various biochemical processes [26]. Thus, although cellular changes caused by high NaCl may generally be due to hypertonicity, this is not necessarily the case. For this reason, to ascribe the effects observed to hyperosmolarity, we investigated hyperosmolarity with mannitol, which is a poorly permeating organic solute [26].

In preliminary experiments, we tested two osmolarity values (500 and 600 mOsm/L) and the hyperosmotic treatment at 600 mOsm/L induced cellular death within 24 h, with the consequent inability of cells to adapt to this very high osmolarity; therefore, this condition was not further analyzed.

Upon 500 mOsm/L hyperosmolarity, the higher levels of NO release within 30 min compared to controls may testify a status of oxidative stress induced by the change in osmolarity.

It is known that NO is an important modulator of intestinal cell functions and signalling under physiological and physio-pathological conditions, and NO can mediate positive or negative effects depending on its relative levels [27].

In our study, betaine supplementation proved to attenuate the hyperosmotic stress condition within 24 h, which was associated with a significant reduction in NO and a positive effect on gene expression of tight junction proteins.

Our data demonstrates that IPEC-J2 cells treated with the 500 mOsm/L medium obtained with mannitol upregulate the expression of osmolyte transporters (*TauT*, *SMIT* and *BGT1*). Studies conducted in tissues usually exposed to osmolarity variations, such as those of the kidney [26], cartilage [28,29,30], brain [31], skin cells [32,33], and liver [34,35], demonstrated that during adaptation to hyperosmolarity, organic osmolytes are transported into cells via specific transporters such as ATA2 for neutral amino acids, BGT1 for betaine, *SMIT* for myo-inositol and *TauT* for taurine [36], whose encoding genes have been defined as osmosensitive genes since their expression can be upregulated by an hypertonic environment [37].

When exposed to hyperosmolar stress, cells increase the gene expression of osmolyte transporters to replace the cellular accumulation of inorganic ions and preserve the cell volume and water content [38,39]. These osmolytes play a role in volume homeostasis, protection against oxidation [12], and different cell injuries [13,14].

In our study, the induction of the three osmolyte transporters was different at different time points, indicating a different pattern of expression as also observed in other tissues such as those of the brain and kidney [37,40]. It was found that hyperosmolarity as well as TNFα can induce a time-dependent decrease in *TauT* and *SMIT* transporters in vitro [40]. Therefore, an inflammatory condition which lasts over time may acutely induce and then reduce the expression and function of these two osmolyte transporters in IPEC-J2 intestinal cells.

In accordance with other authors [7,8] who detected an increase in *IL8* in human intestinal cell lines (HT-29 and Caco-2), we observed that IPEC-J2 hyperosmolar treatment increased the gene expression of the inflammatory cytokines *IL6*, *IL8*, and *TNFα*. This increase is considered as a stress condition that, in humans, can be associated with the onset and progression/exacerbation of several intestinal diseases (e.g., inflammatory bowel disease [IBD], Crohn’s disease, necrotizing enterocolitis, celiac disease) [41,42]. Pro-inflammatory cytokine upregulation can reduce TJ organization and TEER, which are related to the alteration of intestinal cell permeability and barrier functions.

In addition, it was evaluated whether the hyperosmolar treatment had any effect on gene expression of the TJ proteins *occludin*, *ZO-1*, and *claudin 4*, since changes in the cellular architecture can influence these proteins, which are fundamental for the maintenance of the barrier function through intercellular interactions [16]. In particular, we observed a decrease in TJ protein expression concomitantly with the decrease in TEER and increase in pro-inflammatory cytokine gene expression. The inflammatory condition in our study could have negatively influenced the intestinal barrier and, in particular, the TJ proteins responsible for cell-to-cell communication. It was demonstrated that the mucosal barrier and TJ protein integrity in human intestinal cells (T84 and Caco-2) is affected by pro-inflammatory cytokines such as *TNFα*, *IL8*, and IFN-γ [7,43].

Betaine is a natural compound widely present in animals, plants, and microorganisms which contains three methyl groups and, thus, it is a methyl donor and enters into the methionine–homocysteine cycle [10]. When added to the diet of growing pigs, numerous positive effects on pig production performances, meat quality, and reproductive performances have been demonstrated [44,45]. In particular, it has been reported that betaine acts as an effective osmolyte to protect cells from damage caused by hyperosmotic stress [4] and can influence the availability of other amino acids acting as osmolytes under this condition such as glycine and glutamine [46]: in fact, glutamine and glutamate, and their transport, were proven to exert beneficial effects on porcine intestinal cells [47,48,49,50,51]. Therefore, we tested whether the addition of betaine to hyperosmolar medium positively affected TJ protein and pro-inflammatory cytokine gene expression induced by hyperosmolarity.

When betaine was added to the medium, *BGT1* transporter at 3 h was higher compared to medium without betaine, likely to rapidly internalize betaine to compensate for the volume modification and higher amino acids concentration. Consequently, the gene expression of *SMIT* and *TauT* in the presence of betaine decreased at 6 h, as the compensation effect of betaine could exert a negative feedback on the expression of the two transporters.

The gene expression upregulation of *IL6*, *TNFα*, and *IL8* following hyperosmolar treatment [8] was reduced by betaine addition to the hyperosmolar medium. Betaine was proven to be able to increase TJ protein expression, which was reduced by hyperosmolarity. Similarly, it was demonstrated that betaine promotes intestinal barrier function by the up-regulation of *occludin* and *claudin-1*, impaired by lipopolysaccharide (LPS) treatment [52]. Our findings support the positive effects of compatible osmolyte addition observed both in in vitro studies [4,52] and in in vivo studies [44,53,54].

## 5. Conclusions

This study demonstrates the expression of osmolyte transporters in porcine intestinal epithelial cells aimed at allowing for the entry of biocompatible osmolytes, since their role is very important in balancing intracellular osmolarity. Furthermore, betaine treatment counteracts the negative effects of hyperosmolarity in terms of pro-inflammatory and barrier function markers. Further studies are therefore needed to improve our understanding of the effects of molecules that can act as osmolytes. These investigations could allow us to identify what compounds positively influence intestinal transport and could be utilized to develop new therapies against intestinal diseases.

## Figures and Tables

**Table 1:** Target genes and primer sequences used for SYBR^®^ Green qPCR. *18S rRNA* was used as the endogenous reference gene. *TauT*: taurine transporter; *BGT1*: betaine/GABA transporter 1; *SMIT*: sodium/myo-inositol transporter; *IL6*: interleukin 6; *IL8*: interleukin 8; *TNFα*: tumour necrosis factor alpha; *CLDN4*: claudin 4; *OCLN*: occludin; *ZO-1*: zonula occludens 1; rRNA: ribosomal RNA; F: forward primer; R: reverse primer; bp: base pairs.

Target Gene	GenBank Accession nr	Primer Sequence	Amplicon Length (bp)
*TauT* (*SLC6A6*) [20]	NM_001319102	F 5′-CTCAGCACCACCAACTTCACCTC-3′ R 5′-AGCAGACCAGCCAGACCAGAAG-3′	139
*BGT1* (*SLC6A12*) (this study)	XM_021092484.1	F 5′-ACAACTGCTACAGGGACTGC-3′ R 5′-ATCATGGTCACAGCCTTGGG-3′	184
*SMIT* (*SLC5A3*) (this study)	XM_005657149.3	F 5′-GATTCTGAAAGCCATGCAGCG-3′ R 5′-GGTGCTGGAGGAGAAATTCTGA-3′	81
*TNFα* [21]	NM_214022	F 5′-ACTGCACTTCGAGGTTATCGG-3′ R 5′-GGCGACGGGCTTATCTGA-3′	118
*IL6* [21]	NM_214399	F 5′-GGCAAAAGGGAAAGAATCCAG-3′ R 5′-CGTTCTGTGACTGCAGCTTATCC-3′	87
*IL8* [22]	NM_213867	F 5′-CCGTGTCAACATGACTTCCAA-3′ R 5′-GCCTCACAGAGAGCTGCAGAA-3′	75
*CLDN4* [23]	AB235916	F 5′-TATCATCCTGGCCGTGCTA-3′ R 5′-CATCATCCACGCAGTTGGT-3′	71
*OCLN* [24]	FN400888	F 5′-GGAGTGATTCGGATTCTGTCTATGCT-3′ R 5′-CGCCTGGGCTGTTGGGTTGA-3′	423
*ZO-1* [24]	XM_021098896	F 5′-GGCGCACGGCGAAGGTAATT-3′ R 5′-CTATCAAACTCAGGAGGCGGCACT-3′	405
*18S rRNA* [25]	AY265350.1	F 5′-CCCACGGAATCGAGAAAGAG-3′ R 5′-TTGACGGAAGGGCACCA-3′	125

**Table 2:** TEER measurement in IPEC-J2 cells at 10 min, 30 min, 3 h, 6 h and 24 h of the hyperosmolar treatment (500 mOsm/L) obtained with mannitol (Mann) without/with betaine (BET) compared to the control medium (C). Each value represents the mean ± SD of six wells of three independent experiments. # hashtags indicate a statistical difference between each treatment and the control condition (C) at the same time point (*p* < 0.05); * asterisks indicate a statistical difference between each time point and before treatment upon the same treatment (*p* < 0.05).

TEER (%)	10 min	30 min	3 h	6 h	24 h
C	100.00 ± 14.90	99.39 ± 20.31	99.29 ± 19.67	99.24 ± 11.74	86.71 ± 20.23
C + BET	115.22 ± 11.50	112.40 ± 21.64	114.55 ± 16.27	110.15 ± 21.43	115.27 ± 21.49 #
Mann	112.04 ± 8.03	108.31 ± 9.01	102.15 ± 11.06	92.02 ± 9.03 *	57.81 ± 18.13 *#
Mann + BET	123.85 ± 12.36 #	119.84 ± 10.9 #	118.84 ± 17.23 #	117.99 ± 21.49 #	90.38 ± 11.58 *

**Table 3:** NO release (quantified as nitrite, NO_2_^−^) in the supernatants of IPEC-J2 cells at 10 min, 30 min, 3 h, 6 h and 24 h of the hyperosmolar treatment (500 mOsm/L) obtained with mannitol (Mann) without/with betaine (BET) compared to the control medium (C). Each value represents the mean ± SD of six wells of three independent experiments. # hashtags indicate a statistical difference between each treatment and the control condition (C) at the same time point (*p* < 0.05); * asterisks indicate a statistical difference between each time point and 10 min treatment upon the same treatment (*p* < 0.05).

Nitrite (μM)	10 min	30 min	3 h	6 h	24 h
C	1.63 ± 0.18	1.35 ± 0.36	1.63 ± 0.19	1.58 ± 0.11	1.67 ± 0.20
C + BET	1.31 ± 0.11 #	0.96 ± 0.39 #	0.98 ± 0.34 #	1.31 ± 0.21 #	1.08 ± 0.22 #
Mann	2.93 ± 0.08 #	2.08 ± 0.09 *#	1.58 ± 0.27 *	1.08 ± 0.31 *#	1.26 ± 0.08 *#
Mann + BET	2.66 ± 0.29 #	1.85 ± 0.37 *#	1.04 ± 0.16 *#	0.85 ± 0.20 *#	0.81 ± 0.24 *#

## Data Availability

All data are available from the corresponding author upon reasonable request.

## References

[1] Brandtzaeg P. (2011). The gut as communicator between environment and host: Immunological consequences. Eur. J. Pharmacol..

[2] Faria A.M., Gomes-Santos A.C., Gonçalves J.L., Moreira T.G., Medeiros S.R., Dourado L.P., Cara D.C. (2013). Food components and the immune system: From tonic agents to allergens. Front. Immunol..

[3] Cao S., Wang C., Yan J., Li X., Wen J., Hu C. (2020). Curcumin ameliorates oxidative stress-induced intestinal barrier injury and mitochondrial damage by promoting Parkin dependent mitophagy through AMPK-TFEB signal pathway. Free Radic. Biol. Med..

[4] Xu S., Lu S., Wang H., Li S., Feng J. (2021). Protective effect and mechanism of betaine against hyperosmotic stress in porcine intestinal epithelium. J. Funct. Foods.

[5] Taylor C.T., Dzus A.L., Colgan S.P. (1998). Autocrine regulation of epithelial permeability by hypoxia: Role for polarized release of tumor necrosis factor alpha. Gastroenterology.

[6] Xu D.Z., Lu Q., Kubicka R., Deitch E.A. (1999). The effect of hypoxia/reoxygenation on the cellular function of intestinal epithelial cells. J. Trauma.

[7] Grauso M., Lan A., Andriamihaja M., Bouillaud F., Blachier F. (2019). Hyperosmolar environment and intestinal epithelial cells: Impact on mitochondrial oxygen consumption, proliferation, and barrier function in vitro. Sci. Rep..

[8] Németh Z.H., Deitch E.A., Szabó C., Haskó G. (2002). Hyperosmotic stress induces nuclear factor-kappaB activation and interleukin-8 production in human intestinal epithelial cells. Am. J. Pathol..

[9] Torres-Medina A., Schlafer D.H., Mebus C.A. (1985). Rotaviral and coronaviral diarrhea. Vet. Clin. N. Am. Food Anim. Pract..

[10] Wang H., Li S., Fang S., Yang X., Feng J. (2018). Betaine improves intestinal functions by enhancing digestive enzymes, ameliorating intestinal morphology, and enriching intestinal microbiota in high-salt stressed rats. Nutrients.

[11] Wang H., Li S., Xu S., Feng J. (2020). Betaine improves growth performance by increasing digestive enzymes activities, and enhancing intestinal structure of weaned piglets. Anim. Feed Sci. Technol..

[12] Kwon E.D., Jung K.Y., Edsall L.C., Kim H.Y., García-Pérez A., Burg M.B. (1995). Osmotic regulation of synthesis of glycerophosphocholine from phosphatidylcholine in MDCK cells. Am. J. Physiol..

[13] Cortez L., Sim V. (2014). The therapeutic potential of chemical chaperones in protein folding diseases. Prion.

[14] Tao Y.X., Conn P.M. (2018). Pharmacoperones as Novel Therapeutics for Diverse Protein Conformational Diseases. Physiol. Rev..

[15] Krugliak P., Hollander D., Schlaepfer C.C., Nguyen H., Ma T.Y. (1994). Mechanisms and sites of mannitol permeability of small and large intestine in the rat. Dig. Dis. Sci..

[16] Vergauwen H., Verhoeckx K., Cotter P., López-Expósito I., Kleiveland C., Lea T., Mackie A., Requena T., Swiatecka D., Wichers H. (2015). The IPEC-J2 Cell Line. The Impact of Food Bioactives on Health: In Vitro and Ex Vivo Models.

[17] Andrani M., Ferrari L., Borghetti P., Cavalli V., De Angelis E., Ravanetti F., Dall’Olio E., Martelli P., Saleri R. (2024). Short-chain fatty acids modulate the IPEC-J2 cell response to pathogenic E. coli LPS-activated PBMC. Res. Vet. Sci..

[18] Livak K.J., Schmittgen T.D. (2001). Analysis of relative gene expression data using real-time quantitative PCR and the 2(-Delta Delta C(T)) Method. Methods.

[19] Peterson L.W., Artis D. (2014). Intestinal epithelial cells: Regulators of barrier function and immune homeostasis. Nat. Rev. Immunol..

[20] Xia C., Zhang X., Zhang Y., Li J., Xing H. (2021). Ammonia exposure causes the disruption of the solute carrier family gene network in pigs. Ecotoxicol. Environ. Saf..

[21] Meissonnier G.M., Pinton P., Laffitte J., Cossalter A.M., Gong Y.Y., Wild C.P., Bertin G., Galtier P., Oswald I.P. (2008). Immunotoxicity of aflatoxin B1: Impairment of the cell-mediated response to vaccine antigen and modulation of cytokine expression. Toxicol. Appl. Pharmacol..

[22] Royaee A.R., Husmann R.J., Dawson H.D., Calzada-Nova G., Schnitzlein W.M., Zuckermann F.A., Lunney J.K. (2004). Deciphering the involvement of innate immune factors in the development of the host response to PRRSV vaccination. Vet. Immunol. Immunopathol..

[23] Mariani V., Palermo S., Fiorentini S., Lanubile A., Giuffra E. (2009). Gene expression study of two widely used pig intestinal epithelial cell lines: IPEC-J2 and IPI-2I. Vet. Immunol. Immunopathol..

[24] Zou Y., Xiang Q., Wang J., Peng J., Wei H. (2016). Oregano essential oil improves intestinal morphology and expression of tight junction proteins associated with modulation of selected intestinal bacteria and immune status in a pig model. BioMed Res. Int..

[25] Wang S., Wang B., He H., Sun A., Guo C. (2018). A new set of reference housekeeping genes for the normalization RT-qPCR data from the intestine of piglets during weaning. PLoS ONE.

[26] Burg M.B., Ferraris J.D., Dmitrieva N.I. (2007). Cellular response to hyperosmotic stresses. Physiol. Rev..

[27] Mu K., Yu S., Kitts D.D. (2019). The role of nitric oxide in regulating intestinal redox status and intestinal epithelial cell functionality. Int. J. Mol. Sci..

[28] Petronini P.G., De Angelis E.M., Borghetti P., Borghetti A.F., Wheeler K.P. (1992). Modulation by betaine of cellular responses to osmotic stress. Biochem. J..

[29] De Angelis E., Petronini P.G., Borghetti P., Borghetti A.F., Wheeler K.P. (1999). Induction of betaine-gamma-aminobutyric acid transport activity in porcine chondrocytes exposed to hypertonicity. J. Physiol..

[30] De Angelis E., Barilli A., Saleri R., Rotoli B.M., Ravanetti F., Ferrari F., Ferrari L., Martelli P., Dall’Asta V., Borghetti P. (2023). Osmolarity modulates the de-differentiation of horse articular chondrocytes during cell expansion in vitro: Implications for tissue engineering in cartilage repair. Vet. Res. Commun..

[31] Häussinger D., Warskulat U., Schliess F. (1997). Osmosignalling and osmolytes in liver and astrocytes. Adv. Exp. Med. Biol..

[32] Sougrat R., Morand M., Gondran C., Barré P., Gobin R., Bonté F., Dumas M., Verbavatz J.M. (2002). Functional expression of AQP3 in human skin epidermis and reconstructed epidermis. J. Investig. Dermatol..

[33] Warskulat U., Reinen A., Grether-Beck S., Krutmann J., Häussinger D. (2004). The osmolyte strategy of normal human keratinocytes in maintaining cell homeostasis. J. Investig. Dermatol..

[34] Warskulat U., Zhang F., Häussinger D. (1997). Taurine is an osmolyte in rat liver macrophages (Kupffer cells). J. Hepatol..

[35] Weik C., Warskulat U., Bode J., Peters-Regehr T., Häussinger D. (1998). Compatible organic osmolytes in rat liver sinusoidal endothelial cells. Hepatology.

[36] Burg M.B., Kwon E.D., Kültz D. (1997). Regulation of gene expression by hypertonicity. Annu. Rev. Physiol..

[37] Bitoun M., Tappaz M. (2000). Gene expression of taurine transporter and taurine biosynthetic enzymes in brain of rats with acute or chronic hyperosmotic plasma. A comparative study with gene expression of myo-inositol transporter, betaine transporter and sorbitol biosynthetic enzyme. Brain Res. Mol. Brain Res..

[38] Dall’Asta V., Bussolati O., Sala R., Parolari A., Alamanni F., Biglioli P., Gazzola G.C. (1999). Amino acids are compatible osmolytes for volume recovery after hypertonic shrinkage in vascular endothelial cells. Am. J. Physiol..

[39] Alfieri R.R., Cavazzoni A., Petronini P.G., Bonelli M.A., Caccamo A.E., Borghetti A.F., Wheeler K.P. (2002). Compatible osmolytes modulate the response of porcine endothelial cells to hypertonicity and protect them from apoptosis. J. Physiol..

[40] Oenarto J., Görg B., Moos M., Bidmon H.J., Häussinger D. (2014). Expression of organic osmolyte transporters in cultured rat astrocytes and rat and human cerebral cortex. Arch. Biochem. Biophys..

[41] Hubert A., Cauliez B., Chedeville A., Husson A., Lavoinne A. (2004). Osmotic stress, a proinflammatory signal in Caco-2 cells. Biochimie.

[42] Schwartz L., Guais A., Pooya M., Abolhassani M. (2009). Is inflammation a consequence of extracellular hyperosmolarity?. J. Inflamm..

[43] Bruewer M., Luegering A., Kucharzik T., Parkos C.A., Madara J.L., Hopkins A.M., Nusrat A. (2003). Proinflammatory cytokines disrupt epithelial barrier function by apoptosis-independent mechanisms. J. Immunol..

[44] Fu R., Wang Q., Kong C., Liu K., Si H., Sui S. (2022). Mechanism of action and the uses betaine in pig production. J. Anim. Physiol. Anim. Nutr..

[45] Wang B., Wu G., Zhou Z., Dai Z., Sun Y., Ji Y., Li W., Wang W., Liu C., Han F. (2015). Glutamine and intestinal barrier function. Amino Acids.

[46] Knight L.S., Piibe Q., Lambie I., Perkins C., Yancey P.H. (2017). Betaine in the Brain: Characterization of Betaine Uptake, its Influence on Other Osmolytes and its Potential Role in Neuroprotection from Osmotic Stress. Neurochem. Res..

[47] Xi P., Jiang Z., Dai Z., Li X., Yao K., Zheng C., Lin Y., Wang J., Wu G. (2012). Regulation of protein turnover by L-glutamine in porcine intestinal epithelial cells. J. Nutr. Biochem..

[48] Jiao N., Wu Z., Ji Y., Wang B., Dai Z., Wu G. (2015). L-Glutamate Enhances Barrier and Antioxidative Functions in Intestinal Porcine Epithelial Cells. J. Nutr..

[49] Ji F.J., Wang L.X., Yang H.S., Hu A., Yin Y.L. (2019). Review: The roles and functions of glutamine on intestinal health and performance of weaning pigs. Animal.

[50] Gu A., Yang L., Wang J., Li J., Shan A. (2021). Protective effect of glutamine and alanyl-glutamine against zearalenone-induced intestinal epithelial barrier dysfunction in IPEC-J2 cells. Res. Vet. Sci..

[51] Zhu M., Lai W., Yao L., Xu E., Chen X., Zhang Y.Y., Li X.G. (2023). Glutamine Regulates Gene Expression Profiles to Increase the Proliferation of Porcine Intestinal Epithelial Cells and the Expansion of Intestinal Stem Cells. Animals.

[52] Wu J., He C., Bu J., Luo Y., Yang S., Ye C., Yu S., He B., Yin Y., Yang X. (2020). Betaine attenuates LPS-induced downregulation of occludin and claudin-1 and restores intestinal barrier function. BMC Vet. Res..

[53] Lawrence B.V., Schinckel A.P., Adeola O., Cera K. (2002). Impact of betaine on pig finishing performance and carcass composition. J. Anim. Sci..

[54] Mendoza S.M., Boyd R.D., Ferket P.R., van Heugten E. (2017). Effects of dietary supplementation of the osmolyte betaine on growing pig performance and serological and hematological indices during thermoneutral and heat-stressed conditions. J. Anim. Sci..

